# Implementation of Paper-Based Materials in Emergency Architecture: Research and Development of Transportable Emergency Cardboard Houses

**DOI:** 10.3390/ma18174134

**Published:** 2025-09-03

**Authors:** Jerzy F. Łątka, Agata Jasiołek, Daria Pawłosik, Anna Karolak, Paweł Niewiadomski, Paweł Noszczyk, Artur Jörgen, Paulina Sołowiej

**Affiliations:** 1 Faculty of Architecture, Wrocław University of Science and Technology, 50-317 Wrocław, Poland; agata.jasiolek@pwr.edu.pl (A.J.); daria.pawlosik@pwr.edu.pl (D.P.); artur.jorgen@pwr.edu.pl (A.J.);; 2 Faculty of Civil Engineering, Wrocław University of Science and Technology, 50-377 Wrocław, Poland; anna.karolak@pwr.edu.pl (A.K.); pawel.niewiadomski@pwr.edu.pl (P.N.); pawel.noszczyk@pwr.edu.pl (P.N.)

**Keywords:** paper in architecture, cardboard structures, emergency architecture, relief shelters

## Abstract

In response to the growing number of forcibly displaced persons caused by natural disasters and conflicts, this study investigates the use of paper-based materials in the design and construction of temporary emergency shelters. The research presents an iterative development of five full-scale prototypes of the TECH (Transportable Emergency Cardboard House) project. The study combines material testing, thermal simulations, and prototyping to evaluate the structural, thermal, and environmental performance of mass-produced paper elements—such as corrugated cardboard, honeycomb panels, and paper tubes—applied in various architectural and climatic contexts. Each TECH prototype was assessed for durability, thermal resistance, and assembly feasibility. Findings confirm that paper-based materials can meet the basic requirements of emergency architecture while maintaining low environmental impact, ease of transport, and low production costs. The results support the feasibility of using cellulose-based components as sustainable alternatives to conventional relief shelters. The study concludes with design guidelines for further development of long-lasting, low-impact housing units adaptable to diverse climate zones and emergency scenarios.

## 1. Introduction

In recent decades, global crises—particularly political conflicts and natural disasters—have significantly increased the number of forcibly displaced persons. Paradoxically, in the constant growth of the global economy, there are more and more people who find themselves in a state of homelessness.

As the United Nations High Commissioner for Refugees and United Nation Refugee Agency states in the report “Global trends. Forced Displacement in 2024”, there were 123.2 million forcibly displaced people (FDPs) at the end of 2024 due to the persecutions, conflict, violence, and human rights violation or events that seriously disturbed public order [1].

In parallel, natural disasters such as floods, storms, and earthquakes displaced over 45.8 million people in 2024 alone—more than twice the number displaced by conflict, which amounted to 20.1 million people [2]. However, this number might be underestimated. Natural disasters are often perceived to cause only short-term displacement but in reality, they frequently lead to protracted situations.

### 1.1. Shelters

Sheltering, next to water supply, nutrition, sanitation, and healthcare is one of the most important elements that should be provided to FDPs.

**Figure 1:**
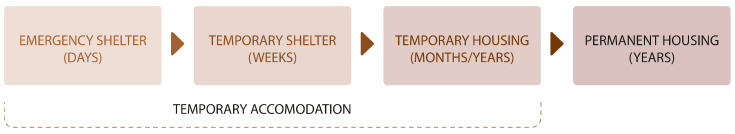
Shelter typology according to UNHCR, classified by intended duration of use.

Among the recognized shelter classifications, the UNHCR model focuses on the intended duration and permanence of shelter types (see Figure 1) [3]:

- **Emergency shelter**: A shelter used at the highest peak of an emergency situation. It can be a private house or a public shelter. This shelter should be used for days as disaster aftermath.
- **Temporary shelter**: A place accommodated for a short time up to several weeks after the disaster. It can be in the form of gathering in public spaces such as a gymnasium or deployable shelter, e.g., tent.
- **Temporary housing**: A more durable space, inhabitable, where victims of natural or man-made disasters can experience resilience. This space provides accommodation from 6 months to up to 3 years. It should provide basic comfort, services, and a sense of security.
- **Permanent housing**: A permanent house where victims of disasters can resettle or return to their original place of stay.

A complementary classification formulated by the International Federation of Red Cross and Red Crescent Societies (IFRCRCS), highlights the potential for shelter evolution through phases of adaptation and upgrading [4].

As a result, an increasing proportion of forcibly displaced individuals are compelled to inhabit makeshift and inadequate conditions for extended periods. In practice, shelters initially designed as temporary solutions—often intended to last only several weeks or months—remain in use for many years, serving as permanent dwellings despite not being designed or equipped to fulfill such a role [5,6,7].

### 1.2. Environmental Impact of Contemporary Emergency Shelters

Increasing attention is being directed toward the environmental and economic implications of post-disaster temporary housing. The sustainability of such shelters is typically evaluated across three principal dimensions: economic viability, environmental responsibility, and social impact. Of these, the environmental dimension presents pressing concerns due to the extensive resource consumption, greenhouse gas emissions, and reliance on non-renewable materials associated with large-scale shelter production [8].

The manufacture of conventional construction materials—such as concrete, steel, and synthetic polymers—entails significant environmental costs. These include high CO_2_ emissions, substantial water consumption, and the requirement for long-distance transportation, all of which contribute to an increased carbon footprint across the shelter’s life cycle [9].

Adopting circular construction strategies—such as the use of regenerated, reused, or recycled materials, modular components designed for disassembly, and self-construction enabled through community involvement—can substantially reduce the ecological burden of temporary housing [10]. Rapidly deployable systems that emphasize waste minimization and reusability support both the functional and environmental objectives of emergency architecture.

A comprehensive sustainable design strategy should be implemented to reduce the environmental footprint across all phases of a shelter’s life cycle: from raw material extraction and manufacturing to transportation, use, and final disposal [11].

Emergency architecture can have significant environmental consequences, particularly through high material consumption and waste. However, by incorporating reuse, modular, and sustainable design strategies, these negative impacts can be substantially reduced. The integration of temporary architecture and reuse within the broader goals of sustainability offers a critical pathway to more environmentally responsible post-disaster recovery efforts [12,13].

Nevertheless, a significant proportion of materials currently used in temporary shelters are not suitable for recycling or reuse at the end of their service life [14]. In many cases materials such as concrete and steel are implemented into the temporary shelters, despite their relatively short-life span. Depending on the scenario, the concrete block or reinforced concrete frame filled with bricks, steel sheets, or metal containers are implemented [15]. The other option is locally available or grown raw materials such as brushwood, timber, straw, and bamboo. Such solutions require appropriate preparation and processing of local materials which are strengthened and improved with the help of tarpaulin, thatch, or corrugated iron [16,17]. This contributes to large volumes of construction waste, presenting challenges for disposal and straining local waste management systems.

Given these constraints, it is crucial to prioritize construction technologies and material systems that minimize environmental impact across the shelter life cycle. Key strategies include prefabrication, modular design, local material sourcing, and recyclability at end-of-life to support a resilient and sustainable post-disaster housing response.

Building upon these general considerations, the following section presents selected case studies of standard humanitarian shelter units, detailing their material composition and verified environmental performance if available. This approach enables a direct comparison of the life cycle impacts of widely adopted systems, such as textile-based tents and modular timber or hybrid timber–steel structures.

### 1.3. Selected Case Studies of Temporary Housing Solutions

Emergency shelters employ a wide spectrum of construction materials, each exhibiting distinct environmental and mechanical properties. According to the UNHCR [17] and the comparative life cycle assessment analysis [14], commonly implemented systems include textile membrane tents, timber-frame modules, and mixed timber–steel frame units. While the projects documented in the UNHCR catalog are not identical to those assessed in the 2022 article, they are technologically comparable in terms of material composition and functional performance, enabling a meaningful alignment of their environmental and technical profiles. Within this context, it is instructive to examine representative shelter types illustrating the breadth of approaches adopted in humanitarian practice—from lightweight, rapidly deployable units to more durable modular systems. Three examples of temporary accommodation, each characterized by distinct technical and material solutions, illustrate key milestones in the evolution of emergency architecture. The first example, the UNHCR Family Tent, represents the most affordable, rapid, and widely implemented global solution, relying on lightweight fabric structures for immediate deployment. The second, the Refugee Housing Unit (RHU), was developed through an international collaborative research process and utilizes prefabricated building components that can be assembled in a manner similar to flat-pack furniture, offering a quick, lightweight, and easily transportable shelter option. The third example is a pioneering approach in which paper is employed as the primary construction material, demonstrating the potential of unconventional, renewable resources in the design of humanitarian housing.

#### 1.3.1. UNHCR Family Tent (FT)

**Figure 2:**
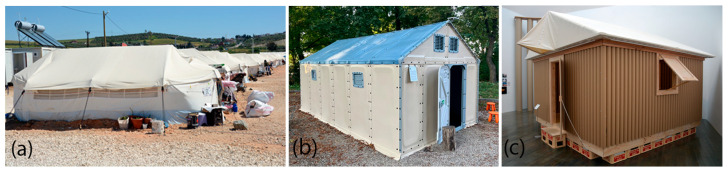
Temporary houses: (**a**) UNHCR Family Tent, (**b**) Refugee Housing Unit, (**c**) Paper Log House.

The most common emergency shelter is UNHCR Family Tent (see Figure 2a), which was designed in both a standard- and an eco-design version. It provides a basic accommodation of 16 m^2^ floor area plus two vestibules, which gives a total area of 23 m^2^. Its outer dimensions are 6.6 m in length, 4 m in width, and 2.2 m in height in the center of the tent. It is suitable for a family of five as the recommended minimum living space per person ranges from 3.5 m^2^ to 4.5 m^2^ in cold climates. The structures consist of a metal pipe frame covered with polyester/cotton roof, wall, and inner tent canvas. In the standard unit configuration, the frame is made of aluminum or galvanized steel tubing, and the outer membrane may also be made entirely of polyester with waterproof coatings [17]. The walls at the bottom part are additionally covered with high-density polythene mud-flaps. The floor is made of polyethene fabric. The tents can have one or two layers of fabric, or can be “winterized”, e.g., have an additional lining made of cotton, a fly sheet, insulating mats, floor protection, and a hole for the stove pipe. The standard Family Tent has a low embodied mass and emissions of approximately 22–25 kg CO_2_ eq/m^2^, with an embodied energy of 320–350 MJ/m^2^, although its short service life (~3 years) increases cumulative environmental impacts over time. Its thermal insulation is negligible (R < 0.1 m^2^K/W) and airborne sound attenuation is minimal [14]. Its lifespan is estimated to be at least one year. FT can be produced in various places on the globe by following its technical specifications as its structure and materials are simplified. The package with a tent has a dimension of 120 × 40 × 30 cm and weighs approximately 62 kg. It can be assembled by three unqualified people in 30 min [17,18,19].

#### 1.3.2. Refugee Housing Unit (RHU)

Refugee Housing Unit is a temporary shelter created by Better Shelter, Sweden in cooperation with UNHCR and with the support of the Ikea Foundation (see Figure 2b). The usable area of the unit is 17.5 m^2^. Its outer dimensions are 5.7 m long, 3.3 m wide, and 2.8 m in height at the roof ridge. The minimal ceiling height inside is 1.8 m. The structure of the RHU consists of two systems: the inner steel frame structure is composed of ten vertical poles, which are connected to the anchoring system and the frame roof structure. The frame is braced with steel cables. The outer layer is installed on the frame. The wall and roof panels are made of flexible and recyclable 5 mm thick polyolefin sheet with UV barrier on the outside and reinforcement layer on the inside. The RHU Shade Net is an external screen that provides 70% of the solar reflection during the day, and during the night it reduces radiated heat loss. The unit is additionally equipped with a photovoltaic system sufficient for lighting and a phone charger. The modular system allows the combining of units into bigger constellations depending on the family size or required function (storage, distribution, or healthcare point, etc.). The lifespan of the unit is 3 years. The RHU is shipped in two flat packages, similar to the ones for IKEA furniture, equipped with all necessary tools. The weight of one shelter is 161 kg. The assembly process can be managed by four qualified workers within 6 h with the use of basic tools [17,20]. While no LCA data are available for this model in the sources, the use of steel and recyclable polymer panels suggests a moderate environmental footprint relative to shelters constructed from heavier, more energy-intensive materials. Its modular design and potential for multiple deployments over its service life may help reduce per-use material impacts.

The comparative LCA presented by Conzatti et al. includes two shelter types that are directly relevant for benchmarking against the case studies described above.

The Timber Frame Shelter records embodied carbon values of approximately 18–20 kg CO_2_ eq/m^2^ and embodied energy of 250–280 MJ/m^2^, with a service life exceeding 5 years when adequately protected, offering moderate thermal and acoustic performance.

On the other hand, the Mixed Timber–Steel Frame Shelter shows slightly higher embodied carbon (20–22 kg CO_2_ eq/m^2^) and embodied energy (280–310 MJ/m^2^), with increased structural durability and potential for additional insulation, albeit at the cost of more complex end-of-life recycling due to its hybrid composition.

It is worth mentioning that while neither of these designs are identical to the UNHCR Family Tent or the Refugee Housing Unit described above, they provide material and performance profiles that are technologically comparable. The Timber Frame Shelter aligns most closely with the Refugee Housing Unit in terms of durability and enclosure type, whereas the Family Tent corresponds more broadly to the lightweight tent-based systems assessed in the 2022 study.

#### 1.3.3. Paper Log House (PLH)

In addition to systems described above, several humanitarian shelter prototypes have explored the use of paper-based materials, aiming to combine lightweight structures with improved environmental performance. Examples include The Paper Log House, which is an emergency shelter designed by Shigeru Ban for the Vietnamese community living in Kobe, Japan, which was severely affected by the great earthquake in 1995 (see Figure 2c). A total of 27 units were built by volunteers. Each of the units covered an area of 4 m × 4 m. Paper Log Houses were composed of timber floors placed on beer crates filled with sandbag foundations, paper tube walls, and the roof made of PVC membrane stretched on the paper tube frame. The walls were made of 108 mm in diameter and 4 mm wall thick paper tubes placed one next to another. The tubes were connected with self-adhesive sponge tape and stressed with steel rods that run horizontally. The walls were attached to the floors by being slid on the timber pegs. After the assembly, paper tubes were painted with polyurethane-based varnish. One unit was assembled by two to four volunteers within six hours. PLH has become one of the most important relief projects in the world and was pioneering in terms of introducing paper as a building material for relief architecture. Its subsequent versions and modifications were built in disaster-stricken areas (2000 Turkey, 2001 India, 2014 Philippines, 2016 Ecuador, 2019 Kenya, 2022 Pakistan, 2023 Japan, 2023 Morocco, 2023 Turkey, 2023 USA, 2023 Korea) [21,22,23].

Although no verified LCA data are currently available for this specific design, its reliance on renewable, recyclable materials and the minimal use of high-embodied-energy components highlights its potential as a low-impact shelter solution.

### 1.4. Paper as a Building Material

The awareness of the ecological impact of relief shelters seemed to be insufficient in past years. However, due to the large scale of the affected population and the crisis intensity, this issue should be taken into account [24]. The need for mass-produced shelters made of commonly and locally available materials, which have relatively good structural and thermal properties, and at the same time are easy to recycle, suggests looking for new products that can be implemented in shelter production. One such product can be paper and its derivatives. Paper is a material of natural origin, and its main building component, cellulose, is considered almost inexhaustible [25]. Moreover, paper can be multi-ply recycled [26]. The history of paper in architecture reaches ancient China and Japan. However, it is the Japanese architect Shigeru Ban who drew worldwide attention with his designs on paper as a contemporary, innovative, and sustainable building material [27]. Ban, for his experimental designs with the use of paper and his humanitarian approach to architecture, was awarded in 2014 with the Pritzker Prize, recognized as the Nobel Prize for architects [28].

**Figure 3:**
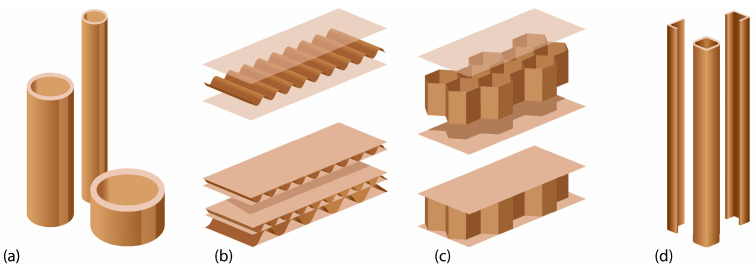
Paper-based materials used as structural building components: (**a**) paper tubes, (**b**) corrugated cardboard, (**c**) honeycomb panel, (**d**) paperboard profiles.

There are several types of paper products that are utilized as building elements (see Figure 3). Those are paper tubes, corrugated cardboard, honeycomb panels, paperboard, and U- and L-shapes made of paperboard. Each of the products is characterized by different structural properties. Paper tubes and U- and L-shape profiles are suitable for linear elements that are subjected to the shear loads, compression or tensile forces, while products such as corrugated cardboard and honeycomb panels are suitable for planar elements, i.e., floor, wall and roof panels. In general, structures, where paper products are used, can be divided into active vector structures (dome or shell system, rod or frame structural system, and their combinations) and active surface systems (panel or plate system) [27].

Apart from numerous structures designed by Shigeru Ban [29], where paper was used as a building material, research was conducted at various scientific institutions and companies with a goal of implementing cellulose-based materials in architecture.

Wikkelhouse (from the Dutch wikkelen, meaning “to wrap”) is a modular prefabricated housing system composed of individual units. Each module measures approximately 1.2 m in depth, 4.6 m in width, and 3.5 m in height. The structural core of each module is created by wrapping 24 layers of corrugated cardboard around a rotating mold, resulting in a self-supporting and well-insulated form. This method adapts packaging production technology to architectural construction. The cardboard core is enclosed within plywood layers, while the external cladding—comprising wood and protective foil—ensures resistance to weather, fire, and mechanical stress. Wikkelhouse is a fully commercialized product currently available on the market [30].

The Full Performance Paper House (FPPH) was developed at TU Darmstadt by the interdisciplinary research group BAMP! (Building with Paper) [31]. Designed in accordance with German building regulations, the project aimed to provide temporary housing solutions for refugees in Germany and for populations in the Global South affected by crises. The FPPH employed a modular system composed of functional segments, each made up of nine sandwich-structured building components. These components comprised multi-layered paper-based materials with an overall thickness of 25–30 cm, primarily using corrugated cardboard and paperboard. The design incorporated various specialized paperboard types, including fire-retardant and high-density variants. For the floor elements, honeycomb cardboard panels were utilized. Polyethylene-coated paper served as the inner moisture barrier, while the exterior was clad in polyethylene-sealed cardboard shingles mounted on timber battens. A full-scale prototype, measuring 3.5 m × 2.5 m × 3.25 m, was constructed on the TU Darmstadt campus for further performance evaluation (FPFH) [32].

Research conducted at Pennsylvania State University investigated the potential of corrugated cardboard waste as a structural and architectural material for low-cost modular housing, as well as for acoustic panels and concrete formwork. The authors proposed a design-to-fabrication grammar system that integrated shape and digital fabrication tools with manual building techniques to enable the creation of customized housing units. The project built on case studies in Asunción, Paraguay, where informal cardboard collectors supplied raw materials. A prototype unit of 42 m^2^ was built using wood-framed wall panels filled with cardboard waste. The design process followed a rule-based grammar, including spatial organization, modular configuration, structural detailing, and fabrication instructions [33,34,35].

ICARO (Innovative Cardboard Architectural Object) was developed by a research team at the University of Catania, Italy, with the aim of creating a prefabricated, modular structure characterized by the use of readily available materials, low cost, lightweight components, and minimal environmental impact. As a full-scale application, the Experience Pavilion (EP) was constructed at the archeological site of Megara Hyblaea in Sicily. The pavilion, measuring 6 m in length, 2 m in width, and 4.5 m in height, was built using prefabricated panels composed of a laminated timber frame filled with triple-wave corrugated cardboard, and externally clad with a ventilated wooden façade [36].

From the mechanical point of view, paper seems to be a promising building material [37,38]. As described above, there are many examples of buildings constructed from paper materials in various systems. As examples, the application of paper tubes as beams or columns in the frame structures [39,40] or corrugated cardboard and honeycomb panels when laminated as walls [41,42,43,44] can be mentioned.

The literature also presents descriptions and results of tests of different paper-based elements in terms of strength.

The research of paper tubes is presented i.a. in [40,45,46,47].

In turn, research of paper products such as honeycomb panels, corrugated paper honeycombs, and honeycomb cardboard is described in [41,44,47,48,49].

And finally, research of corrugated cardboard is presented in [50].

The results of selected experimental tests of paper elements mentioned above are presented below.

The compressive and flexural parameters of paper tubes of various parameters in terms of the geometry (diameters and wall thickness) were described in [39,46]. The results of the compressive strength obtained in the studies ranged from 5.0 to 10.0 MPa, depending on the dimensions. The modulus of elasticity in compression was 1.0 up to 2.0 GPa (the larger the diameter and the thickness of the tube wall, the higher the value). The flexural strength ranged from 8.0 to 16.0 MPa, and the modulus of elasticity in bending was approx. 1.5–2.0 GPa, again depending on the dimensions.

The compressive and tensile strengths of paper tubes described in [47] were approx. 8.0 MPa. The modulus of elasticity for the paper tubes was also determined and it was approx. 1.0–1.5 GPa. In turn, the honeycomb panels with thickness of 20 mm were tested for the flexural strength and they obtained the value of approx. 7.0 MPa and the modulus of elasticity was approx. 1.0 GPa.

Similar results for paper tubes are shown in [49]. The obtained modulus of elasticity under long-term loading was approx. 1.0 GPa and approx. 1.0–1.5 GPa under short-term loading. Furthermore, the flexural strength under short-term loading was approx. 6.6 MPa and the compression strength under short-term loading was approx. 4.4 MPa.

In turn, the results of corrugated paper honeycomb tests presented in [44] were as follows: the out-of-plane compressive strength—1.4 MPa, the out-of-plane compressive modulus—0.2 GPa, the shear strength in a weak direction—0.4 MPa, the shear strength in the strong direction—0.8 MPa, the shear modulus in a weak direction—0.04 GPa, and the shear modulus in strong direction—0.09 GPa.

And finally, the maximum strength obtained in research of corrugated paper honeycomb core sandwich composites presented in [48] was approx. 1.2 MPa.

In recent years, there has also been a growing interest in the use of cellulose-based materials, such as corrugated cardboard and honeycomb, as an alternative ecological thermal insulation materials in construction. More studies on the thermal properties of these materials are available in the literature. Asdrubali et al. [51] conducted experimental and numerical studies of the thermal conductivity of corrugated cardboard panels, obtaining λ values in the range of 0.0493–0.0598 W/mK depending on the type of wave (C and E). Cekon et al. [52] compared the thermal properties of corrugated cardboard and honeycomb, indicating a higher thermal conductivity of the latter (0.0706–0.1167 W/mK). Salavatian et al. [53] confirmed these results by testing 50 mm high honeycomb panels. In a review article, Łątka et al. [37] compiled data from nearly 200 publications, indicating that the thermal conductivity of paper materials is in the range of 0.05–0.15 W/mK. The authors emphasized the importance of air voids with a height of ≤6 mm in limiting convection. Gaudelas et al. [54] studied the effect of wave height (17–19 mm) on thermal conductivity, but without taking into account the humidity or heat capacity of the material. It is also worth noting the research by Gray-Stuart et al. [55], who demonstrated anisotropy of thermal conductivity of paper depending on the fiber direction. Wang et al. [56] obtained the lowest value of λ for solid paper (0.065 W/mK), and Lavrykov et al. [57] reported a range of 0.08–0.18 W/mK for various types of coated solid paper. Issues related to humidity and sorption were analyzed by, among others, Bandyopadhyay et al. [58], Lyngå and Sikö [59], and Gimaker [60], indicating the need to include these parameters in the assessment of thermal properties. Bach [61] in her doctoral dissertation emphasized the ecological potential of paper but limited herself to the analysis of one direction of heat flow. Extensive studies of the influence of the direction of heat flow on the thermal conductivity of corrugated board and honeycomb were conducted by Noszczyk et al. [62]. Tests according to the EN12667:2002 standard [63] in the FOX314 device showed even twice as good (lower) thermal conductivity in the direction perpendicular to the corrugated cardboard wave and air pockets in honeycomb panels.

The above-mentioned research results confirm that different paper-based products achieve appropriate parameters in strength tests and also thermal conductivity as thermal insulation, sufficient for their use in construction purposes.

### 1.5. Objectives of the Study

The increasing global demand for emergency shelters, driven by armed conflicts, natural disasters, and large-scale displacement, has drawn attention to the significant environmental footprint of current temporary housing solutions. Conventional emergency architecture frequently relies on industrial materials with long degradation periods, such as plastics, concrete or steel, or on composites that are difficult to recycle, leading to long-term waste accumulation and resource depletion. While locally sourced materials offer potential benefits in reducing transportation impacts and supporting regional economies, their availability in crisis contexts is often limited due to the time-intensive processes of cultivation, extraction, or manufacturing. In recent years, efforts have been made to explore natural and renewable materials as viable alternatives, with particular interest in cellulose-based products. These materials, derived from abundant and biodegradable sources, demonstrate promising structural, thermal, and environmental performance characteristics that could address both functional and sustainability requirements. Preliminary research and experimental prototypes suggest that cellulose-based solutions may represent a viable pathway toward reducing the environmental impact of emergency shelters while maintaining essential performance standards. Further investigation into their durability, adaptability, and production scalability is therefore warranted to guide future design and deployment strategies.

The objective of this study is to investigate the properties and potential applications of commonly available paper-based materials for use in emergency and relief housing. The research aims to evaluate these materials in relation to structural, thermal, and environmental performance criteria, and to assess their feasibility in meeting the specific needs of displaced or vulnerable populations. In parallel, the study pursues the design, development, and prototyping of deployable shelters that could offer both adequate living conditions and human dignity for users. The implementation of full-scale TECH (Transportable Emergency Cardboard House) prototypes enabled empirical observation of architectural, structural, and material solutions under real-life conditions.

Accordingly, the specific aims of the research are to

(i) design and develop paper-based shelters based on varied design criteria and expected lifespans;
(ii) validate the architectural concept through research by prototyping;
(iii) analyze and compare the performance, strengths, and limitations of each design iteration; and
(iv) formulate recommendations for the further development and implementation of paper-based emergency architecture.

## 2. Materials and Methods

The materials used in this study are mass-produced by the paper industry. These products, i.e., paper tubes, L- and U-shapes made of paperboard, honeycomb panels, and corrugated cardboard, are industrialized products available worldwide. They are most often used for the production of packaging or protective elements for shipments. Therefore, it can be assumed that these products can be manufactured locally from recycled materials, which reduces the need for transport and thus the negative impact on the environment.

**Figure 4:**
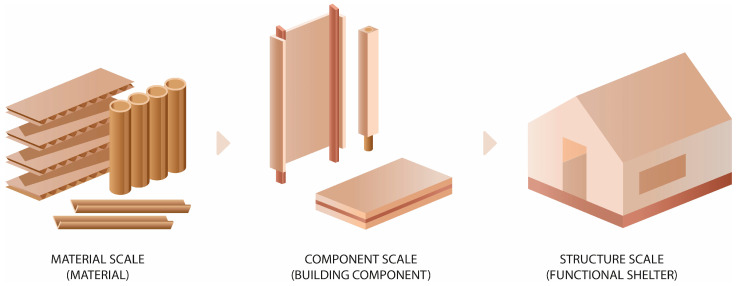
Scale-based framework outlining the stages of the study, from standard paper-based materials, through building components, to complete shelter units.

To ensure clarity and comparability of the material solutions implemented in the temporary housing prototypes, the description of the applied materials is organized according to a three-level scale (see Figure 4):

- **Material scale,** which refers to individual products of cellulosic origin, such as corrugated cardboard, honeycomb panels, paperboard, and paper tubes. These materials are characterized by specific physical and mechanical properties and form the base for further processing.
- **Component scale,** which includes assembled construction components made from the above-mentioned products, such as wall modules, roof panels, beams, and connectors. This level involves the integration of materials through bonding, lamination, folding, or structural reinforcement.
- **Structure scale,** which relates to complete architectural prototypes, composed of selected components assembled into a functional shelters.

The selected materials, combined in various configurations, form prefabricated building components with differing characteristics. These components constitute multiple iterations of the Transportable Emergency Cardboard House (TECH), specifically versions 01 through 05. Accordingly, the paper materials and their properties are detailed in Section 2.1: Materials’ properties, while the individual building components and complete structures are discussed in Section 3: Results and discussion.

### 2.1. Materials’ Properties

The basic mechanical parameters of paper-based products that have been determined in the experimental research [64,65], which have been applied in TECH projects, are presented in the table below (Table 1).

**Figure 5:**
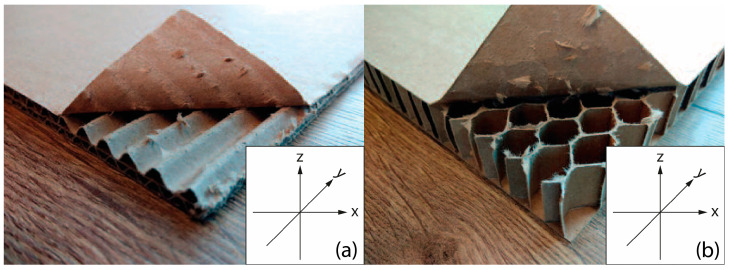
Possible directions of heat flow through the cellulose material: (**a**) corrugated cardboard; (**b**) honeycomb panel.

The basic material parameter in thermal analysis of buildings is the thermal conductivity coefficient (λ). The table below presents a summary of this parameter based on the literature research and our own research [62]. As part of our own research, thermal conductivity measurements were carried out on 77 samples made of corrugated cardboard and honeycomb. The samples were stored in a room with a relative humidity of 30–40% and a temperature of 22–24 °C for 3 months. The thermal conductivity coefficient (λ) was measured at an average sample temperature of 10 °C (lower hotplate 20 °C, upper hotplate 0 °C). Note that the obtained results referred to the vertical (upward) direction of heat flow. The tests were performed using the stationary method with the FOX 314 plate apparatus (TA Instruments, New Castle, DE, USA), in accordance with the PN-EN 12667:2002 standard [63]. For corrugated cardboard, the influence of the wave type (B, BC, E, EB, EE) and the direction of heat flow (Z, Y, X—see Figure 5) was analyzed. For honeycomb, the influence of the height of air gaps (10, 25, 50 mm) and the direction of heat flow was studied. The results showed that the lowest thermal conductivity was obtained for the B-type corrugated board (λ = 0.039 W/mK), while the highest was obtained for the 50 mm high honeycomb (λ = 0.135 W/mK) as shown in the Table 2. An increase in thermal conductivity was also observed with the increase in the height of the air voids, which is caused by the increase in the share of heat flow by convection inside the air voids.

### 2.2. Research Methodology

This study employed a multi-phase research methodology integrating the literature analysis, experimental testing, and iterative prototyping. A comprehensive literature review was conducted to examine the challenges of relief architecture, with a particular focus on temporary housing solutions and their environmental impacts. A separate strand of the review concentrated on paper-based architecture, including both academic studies and implemented commercial examples. The review also covered the mechanical performance of various paper products—such as strength, stiffness, and durability—as well as their thermal insulation properties.

Subsequently, experimental testing was carried out on selected cellulose-based materials. These included mechanical strength assessments and thermal conductivity measurements to evaluate their suitability for structural and insulation applications.

The design phase was conducted using the Research by Design methodology, supported by the Product Development in Architecture (PDA) framework [70]. This iterative process resulted in the development of five design versions of the TECH (Transportable Emergency Cardboard House) prototype, each incorporating revised objectives and performance criteria.

### 2.3. Protytyping Process

Each design iteration culminated in the construction of a physical prototype, which was tested under real-life conditions to verify the proof-of-concept (POC). It enabled validation of key aspects, including the production process, transport logistics, on-site assembly, connection details, material protection methods, and the overall spatial configuration. The POC stage served as a critical checkpoint for assessing the feasibility of full-scale implementation and further development.

This phase was closely linked to the research by prototyping approach, which treats the prototype not merely as a final product, but as an active tool for knowledge generation. Through the physical construction and empirical evaluation of each TECH version, insights were gained regarding the behavior of paper-based materials under load, their durability in varying climatic conditions, and the ergonomic and spatial qualities of the shelter design. Prototyping enabled a tangible assessment of the integration between materials, detailing, and performance, fostering an iterative feedback loop between design intention and technical feasibility. The hands-on experimentation inherent in this approach was essential in uncovering limitations, refining material treatments, and optimizing construction methods for emergency deployment scenarios.

### 2.4. Design Evaluation

The prototyping process allowed for evaluation of the structure feasibility, construction time, and ease of assembly by non-professionals equipped only with basic tools. After the assembly of each prototype, strengths and weaknesses were analyzed and guidelines for the next design iteration were formulated.

Several quantitative indicators were calculated to assess performance of TECH envelopes, which are essential parts of each design, providing structural stability, thermal insulation and protection against weather conditions. The envelope thickness (d) and weight (m) were calculated to assess the general material consumption, thermal resistance (R), and transmittance (U) to evaluate thermal efficiency of the structure and, consequently, the energy demand associated with heating and cooling. The heat of combustion of materials used in the design was calculated as one of the fire safety indicators and paper shares in the materials used as an indicator of pro-environmental characteristics, in particular recyclability and the use of recycled raw materials.

Thermal properties of the envelopes were determined via thermal simulation for stationary heat flow through the partition, assuming −20 °C on the cold side of the partition and +20 °C on the warm side. The following thermal conductivity parameters were assumed for numerical calculations: paper honeycomb 50 mm (0.125 W/mK), paper honeycomb 25 mm (0.095 W/mK), corrugated cardboard BC flute (0.050 W/mK), paperboard (0.25 W/mK), and cellulose (0.038 W/mK). The calculations took into account the thermal resistance of air on both sides of the partition, i.e., Rse = 0.04 and Rsi = 0.13 m^2^K/W. The effectiveness of thermal insulation was evaluated using two key ratios: thermal resistance to envelope thickness (R:d) and thermal resistance to weight per square meter (R:m). The heat of combustion per square meter of the envelope—an important factor influencing the building’s fire load—was determined based on the calorific values of the constituent materials, as specified in the [71].

Furthermore, a range of qualitative parameters was assessed, including level of resistance against fire and water, mechanical damage on external and internal surfaces, as well as price and availability of component and materials used in the envelope design. Resistance levels were rated in a three-point scale (high, medium, and low), based on the general characteristic of coatings, laminates, and cladding materials used in the design, the literature data and technical data sheets in the case of conventional building materials. Price and availability were rated using a three-point scale (good, medium, poor) based on an analysis of the Polish market for paper and construction materials.

Afterwards, the overall performance of the envelope was measured using a Performance Score (PS) [72]. This score represents the aggregate of numerical evaluations across several categories. Each envelope was assigned a score ranging from 0 (indicating the lowest performance) to 2 (indicating the highest performance) for the U-value, and the R:d and R:m ratios. Additionally, scores between 0 and 4 were allocated for material cost, water, fire, and mechanical resistance. Points were assigned according to the following criteria.

- In the category of thermal insulations, envelopes were assessed based on the U-value calculated according to the methodology described in the previous paragraph. For U-values lower than 0.5 W/m^2^K—2 points were assigned, for 0.5 < U < 1.0—1 point, and for U > 1.0—0 pints.
- For R:d (thermal resistance to envelope thickness) ratios, envelopes were assigned 2 points if the ratio was higher than 0.15, 1 point when 0.15 > R:d > 0.10, and 0 point when R:d < 0.10.
- For R:m (thermal resistance to envelope weight) ratios, envelopes were assigned 2 points if the ratio was higher than 0.20, 1 point when 0.20 > R:d > 0.15, and 0 point when R:d < 0.15.
- In the categories of fire, water, and mechanical damage resistance, each envelope was assigned 2 points for high level of resistance on each of its surfaces (external and internal), 1 point for medium resistance, and 0—for low.
- In the categories of price and availability, each envelope was assigned 4 points for good availability, 2 points for medium, and 0—for poor.

The above parameters have been selected in such a way that their range corresponds to the case studies analyzed and effectively demonstrates the differences between them. It should be noted that when applying the PS method to evaluate a different set of designs, it is recommended to adjust the threshold values accordingly.

## 3. Results and Discussion

In this section, the results of the research are presented in the form of TECH projects and prototypes.

There were five iterations of TECH, which differed in terms of design assumptions, formal solutions, type of relief architecture, materials used, connection details, method of protecting cellulose-based materials from external conditions, production method and process, as well as transport capabilities and assembly. The developed TECH units were designed for areas with a temperate climate. The prototypes were operated in European climates, specifically Polish ones.

### 3.1. TECH 01

**Figure 6:**
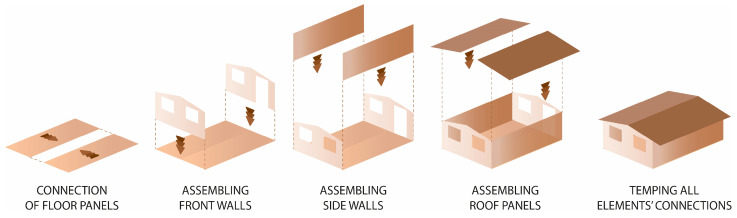
Component assembly sequence developed for the full-scale unit of TECH 01.

The first attempt, i.e., TECH 01 was designed as an emergency shelter in 2014 by Jerzy Łątka. The project was commissioned concerning the growing influx of refugees from the Middle East to Europe. The main aim of the project was to create a low-cost, low-weight, and eco-friendly structure that could be used instead of UNHCR Family Tents with a maximum lifespan of 18 months. The mass production, shipping logistics, and ease of construction were the technical design objectives. The structure should consist of several prefabricated building components that could be shipped to the site in one of the Middle East countries and assembled by non-professionals without the need for special building tools (see Table 3 and Figure 6).

#### 3.1.1. TECH 01—Design and Materials

**Figure 7:**
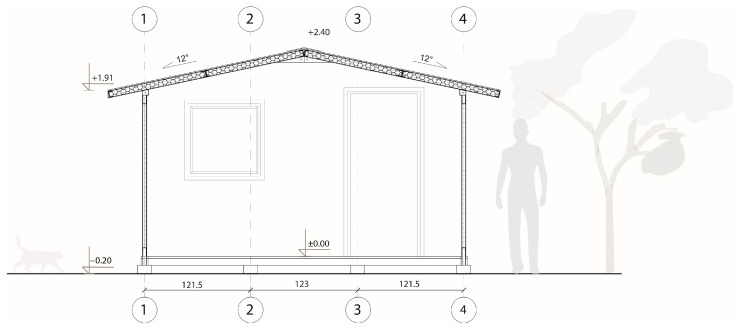
Section of TECH 01.

The unit that covered the usable area of 17.4 m^2^ with a height that varied from 1.9 m to 2.4 m at the ridge was a hybrid rod–panel structural system, i.e., wall and roof panels were reinforced with integrated timber linear elements and thus were load-bearing building components (see Figure 7).

**Figure 8:**
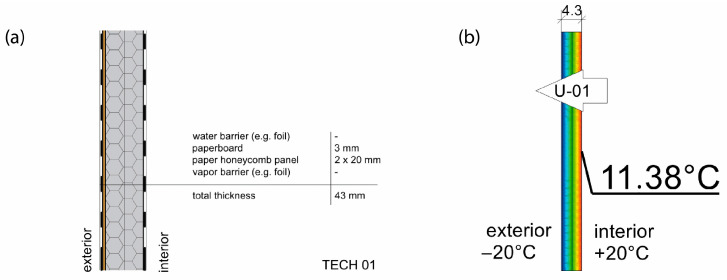
Layer composition applied in the TECH 01 external wall (**a**) and its corresponding numerical simulation of temperature distribution with indicated surface temperature (**b**).

TECH 01 consisted of three types of building components: floor, wall, and roof. The floor component was made of impregnated timber beams (80 mm × 80 mm) covered with an 18 mm thick oriented strand board (OSB). Wall components were made of impregnated cardboard U-shape frames filled with two cardboard honeycomb panels with a thickness of 20 mm each. The walls were covered with vapor barriers from the inside and a protective, waterproof layer from the outside (see Figure 8). Timber joints were integrated into the panels and allowed the wall panels to be connected with slots in the floor components and roof structure. The roof was composed of two prefabricated panels that were connected at the ridge. The simplicity of the structure allowed for assembly by a non-professional crew. As the shelter was made out of paper-based products, it was possible to paint them or laminate with a colored layer to customize shelters and enhance personal identification.

#### 3.1.2. TECH 01—Prototyping and Assembly

**Figure 9:**
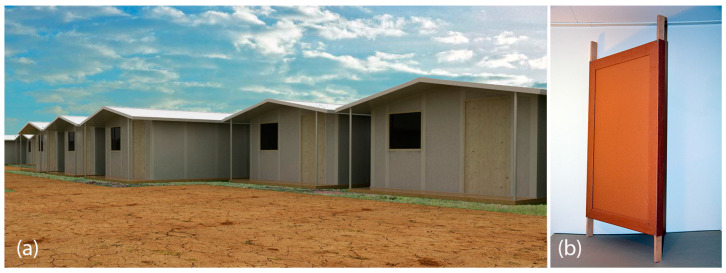
TECH 01 (**a**) visualization of TECH 01 units, (**b**) fabricated external wall component.

There was only a wall panel prototype of TECH 01 (see Figure 9). Therefore, the assembly process was not tested in reality; however, it assumed step by step mounting. First, the timber floor components would be connected using screws and laid on the leveled ground with a foil interlayer. Next, the wall panels would be plugged. The panels would be screwed with the floor and each other. Once all the wall panels are installed, the roof panels are attached (see Figure 6). At the end, the connections between building components would be covered with adhesive tape.

#### 3.1.3. TECH 01—Evaluation

TECH 01 was a design of emergency shelter composed of timber- and paper-based materials. The shelter was designed for hot climates and would serve as a substitute for the UNHCR Family Tent. The materials used and the simplicity of the structure would allow for low-cost and fast production. The framed wall panels should be further evaluated and tested for resistance to the axial and lateral forces such as wind pressure. Inner and outer protection layers of the panels should be tested with the use of different products such as self-adhesive foils that can be removed after using the shelter. The prefabricated building components and their limited weight would allow for easy assembly by non-professionals without the use of special tools. Due to the limited thickness of the wall and roof panels, the shelter would provide limited thermal and acoustic insulation. However, it would give more privacy and comfort than the UNHCR tent. The waterproofing should be tested in real conditions, especially the connections between elements that were secured with self-adhesive tape.

### 3.2. TECH 02

The second version of TECH was designed in 2015 by students of the Faculty of Architecture Delft University of Technology under the supervision of Jerzy Łątka and Marcel Bilow, and a full-scale prototype was built at the university campus (see Table 4).

The design objectives set for TECH 02 were based on the experiences gained during the preparation of TECH 01. Bearing in mind low thermal insulation of TECH 01, direct contact with the ground, and universal shape, which was not suited to certain climate conditions, the goals of the second version were reworked. Additionally, new objectives assumed a cardboard frame structure that would be separately waterproofed.

The anticipated location of the shelter was northern Iraq, therefore the shelter should meet the given climate conditions, where temperature can rise in the summer up to 48 °C and drop in the winter to −11 °C. Both rain and snow were also considered. The assumed lifespan of the shelter was 3 years; therefore, it fell into the category of temporary shelter or temporary housing [73].

#### 3.2.1. TECH 02—Design and Materials

**Figure 10:**
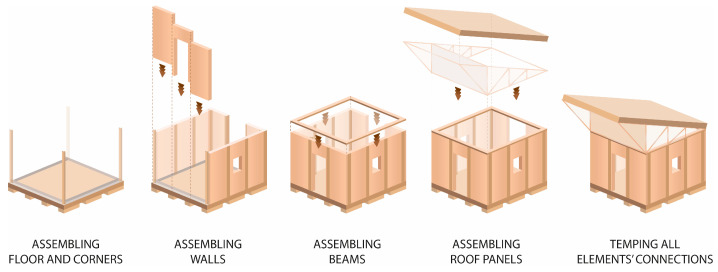
Component assembly sequence developed for the full-scale unit of TECH 02.

TECH 02 was a single-space unit with a usable area of 13 m^2^ intended for a family of three people. It was composed of a floor, wall frame structure, wall panels, roof structure and roof panels. The incorporated structural system was a rod system where pillars, beams, and trusses were the main load-bearing elements (see Figure 10).

**Figure 11:**
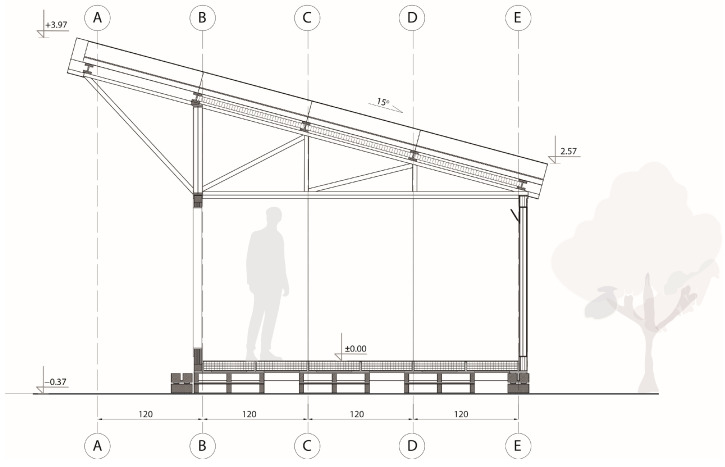
Section of TECH 02.

There were several passive energy-saving solutions incorporated into the project (see Figure 11). Next to the regular wall panels, there was Trombe wall panel elaborated, which provided passive ventilation, heating, and cooling. The roof was designed as a single pitch tropical roof with a Venturi effect with an additional eave to provide a shaded space in front of the house.

**Figure 12:**
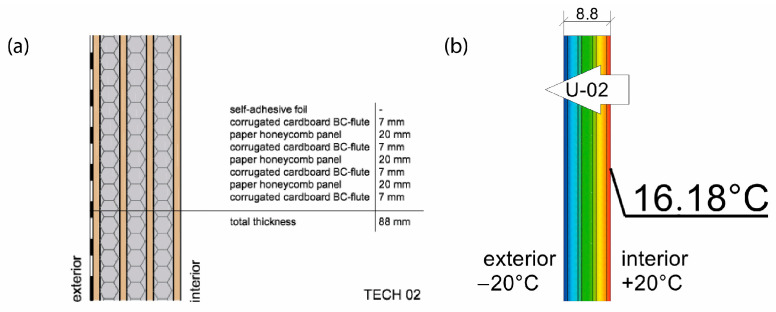
Layer composition applied in the TECH 02 external wall (**a**) and its corresponding numerical simulation of temperature distribution with indicated surface temperature (**b**).

For an extra distance from the ground, the foundation blocks were made of EURO pallets. On top of it, the T-shaped cardboard beams with the 10 mm thickness of the flanges were layered. The beams were covered with 12 cm thick floor sandwich panels made of cardboard honeycomb panels and OSBs. The structure of the walls consists of cardboard beams and pillars as load-bearing elements which were infilled with sandwich cardboard panels with the dimensions of 1.2 by 2.0 m and 63 mm thickness (see Figure 12). The size of the wall panels allowed for fitting the transportation standards. The wall panels were composed of corrugated cardboard and cardboard honeycomb panels. The roof structure consisted of four different elements: trusses, H-beams in a cross direction of the trusses, roof plates, and finishing layer. The trusses and H-beams were made out of U-profiles connected to timber planks. The roof plates were made of corrugated cardboard plates folded into a triangular shape to create ventilation channels for the Venturi effect. The final layer was a waterproof foil. The main structural cardboard elements of the shelter were impregnated with the use of yacht lacquer, and the cuts were additionally protected with the liquid rubber. The wall panels were covered with self-adhesive plastic foil.

#### 3.2.2. TECH 02—Prototyping and Assembly

**Figure 13:**
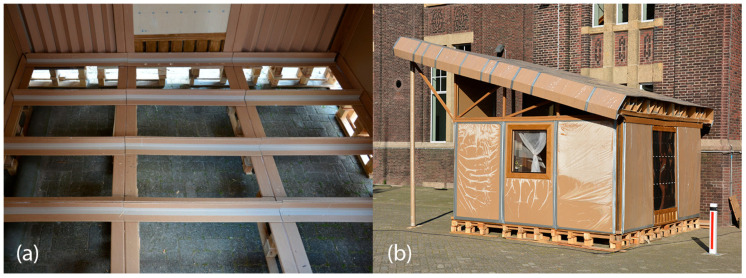
Prototype TECH 02, (**a**) elevated floor structure during the assembly, (**b**) full-scale prototype.

The TECH 02 prototype was built during the Bucky Lab course provided at the Faculty of Architecture Delft University of Technology (see Figure 13). The building process was executed by 13 students within two weeks. The students were divided into groups that simultaneously worked on the floor, wall, and roof. Once all the elements were cut and prepared, the assembly process had started. First, the EURO pallets were placed in two layers to achieve a desired distance from the ground. They were covered with a T-beam grid and sandwich honeycomb and OSB plates. Next, the corner pillars were fixed to the foundation with screws. Subsequently, the wall panels and T-shaped pillars were fixed to the base. Once all the walls were put upright, the U-shaped beams were fixed from above to brace the walls. The roof trusses were fixed to the U-shaped beams and covered with plexiglass plates from the sides and the front of the shelter. Finally, the transverse H-beams were laid down and the corrugated cardboard layer with air channels was placed.

The production of the building elements required the use of professional building tools and power tools, but the assembly process was conducted only with basic drilling machines.

#### 3.2.3. TECH 02—Evaluation

TECH 02 was evaluated as a temporary shelter for victims of warfare in Northern Iraq. The prototype was exposed to natural conditions for six days. The foundation elements made of EURO pallets were a low-cost solution; however, there were too many elements which would cause transportation and assembly problems, especially on a not well-leveled terrain. Moreover, the multipoint contact with the ground might cause the capillary rising of water into the timber and cardboard structure. The mixed frame and paneling wall structure provided sufficient stability, and no extra stiffening was needed. The problem occurred at the corners of the shelter, where the paper-based pillars were exposed to natural conditions including rain. The protective layer made of self-adhesive plastic foil was easy to remove in case of recycling, but on the other hand, the connection to the panels was not efficient. The roof, despite the fact that it corresponded to the local conditions, was complicated in assembly and its structure was not appropriate for the push and pull of the wind. Therefore, alternative roof concepts such as flat, double-pitched, and vault roof could be considered.

### 3.3. TECH 03

This version of TECH was designed as a temporary house for refugees who came to Europe during the great influx, which started in 2015. In TECH 03, several details were improved, including the structure of the roof, foundation, wall panels, the connection between the elements, components size, and impregnation methods. TECH 03 was designed by Jerzy Łątka in cooperation with Julia Schönwälder. The prototype was executed in 2016 by the students from the Wrocław University of Science and Technology under the supervision of Jerzy Łątka and Marcel Bilow (see Table 5).

The goal for TECH 03 was to propose a temporary housing unit for asylum seekers. The unit was intended to be easy to produce and build with the use of paper-based elements. The unit should be adaptable to different spatial and functional layouts, allowing for the accommodation of different groups from small families to large groups of people.

Deriving from the previous project, several elements, such as timber floor structure, exchangeable wall panels, and the size of the roof were changed.

#### 3.3.1. TECH 03—Design and Materials

**Figure 14:**
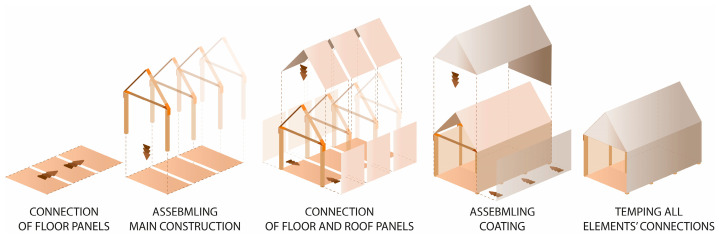
Component assembly sequence developed for the full-scale unit of TECH 03.

**Figure 15:**
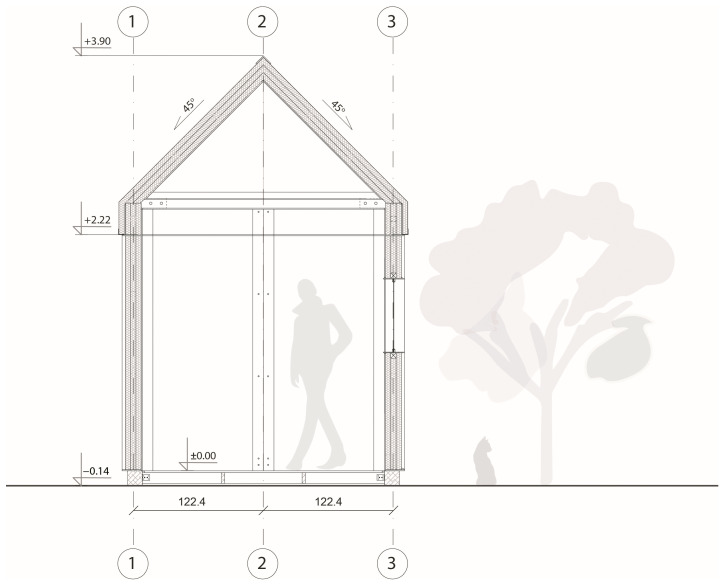
Section of TECH 03.

TECH 03 was a unit with the dimensions of 2.3 m width, 5.7 m length and 3.9 m height. The structure consisted of prefabricated frames infilled with cardboard wall and roof panels (see Figure 14 and Figure 15). The wall and roof panels were inserted into the frame from the outside and screwed, which allows for the replacement of damaged panels, if necessary, without affecting the integrity of the whole structure. The roof was double-pitched at 45°, this allowed for creating an extra storage space at the small mezzanine. The house was equipped with a photovoltaic panel and battery that enabled the house to be lit for 48 h under an overcast sky.

**Figure 16:**
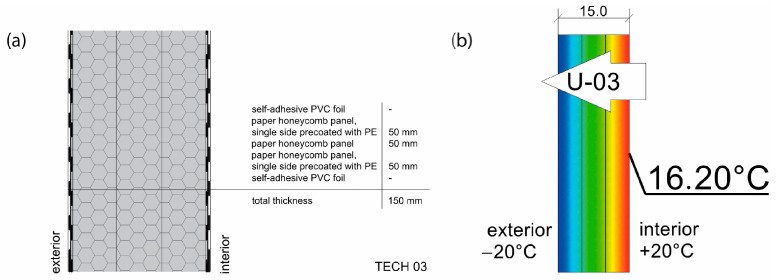
Layer composition applied in the TECH 03 external wall (**a**) and its corresponding numerical simulation of temperature distribution with indicated surface temperature (**b**).

The structure was composed of timber floor and cardboard frame, and wall and roof panels with the dimensions of 120 cm × 240 cm. The frame elements were made of 10 cm × 10 cm paperboard L-shapes with 10 mm thickness of the wall, laminated in the form of T-beams. The T-beams were connected to the floor and each other using plywood joints. The wall and roof panels were made of three layers of 5 cm thick cardboard honeycomb panels (see Figure 16). The round windows were made of paper tubes waterproofed with liquid rubber and the door was a sandwich panel made of plywood and honeycomb. The frame structure elements were impregnated with polyurethane varnish. The building envelope panels were coated with polythene film (during the panels’ production) and additionally covered with PVC foil from the outside.

#### 3.3.2. TECH 03—Prototyping and Assembly

**Figure 17:**
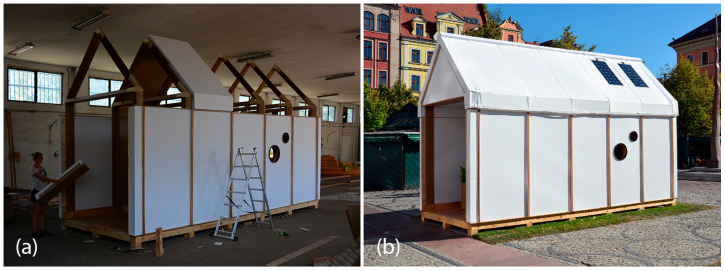
Prototype TECH 03, (**a**) prototype assembly, (**b**) full-scale prototype presented to the public.

The prototype of the unit was built in 2016 in Wrocław during the Summer School of Architecture workshops by a group of ten students (see Figure 17a). The students worked in task groups focusing on floor, frame, panels and window-and-door carpentry. Once the elements were prepared, subsequent frames and panels were fixed. After completion of the walls, the roof panels were attached to the cardboard rafters. Due to the risk of exposure of the cardboard structure to moisture, the whole house was assembled in the workshop and later transported in one piece to the desired location—the main square of the city of Wroclaw, where it was exposed for about a week (see Figure 17b). Next, the house was moved to the campus of WUST, where it stayed for another 1.5 years.

#### 3.3.3. TECH 03—Evaluation

TECH 03 was a proposition of a temporary house made of paper-based elements with a maximum lifespan of three years. The house had a neutral shape and color to fit different European contexts.

During the exposure to the natural conditions, the lowest temperature observed was −10 °C and the highest was 36 °C. The strongest wind reached the speed of 65 km/h, and the highest level of relative humidity was 90%. Thanks to the timber base and frame structure strengthened with panels, the house withstood the forces caused by the wind. After 18 months local damages occurred at the structure. The biggest damage was caused by birds that picked the ridge of the house. Being handmade, this led to imprecise production of some elements and imprecise sealing, causing some leaks around the windows. Even though the structure was erected by non-professionals and the assembly process did not require specialized tools, a large number of elements and connections combined with the height caused production problems. Production in an indoor environment prevented the paper elements from getting wet before the building envelope was enclosed, but such a solution hindered transportation [74].

### 3.4. TECH 04

The fourth edition of Transportable Emergency Cardboard House was developed as a temporary shelter suitable for use in colder climates, with a minimum expected lifespan of 5 years. It was designed in 2018 by Jerzy Łątka and Agata Jasiołek and prototyped by a team of students (see Table 6).

As a result of research and development conducted by the authors on the previous editions, the design criteria set for the TECH 04 focused on improving specific characteristics of the shelter. The shelter should be modular with ease of extension and composition in different spatial arrangements, depending on the scenario and family size. The structural system of the unit should be easy for assembly. The previously proposed systems (TECH 02 and TECH 03) were composed of rod structures filled with floor, wall, and roof panels. In the new project, the system was simplified and delivered with the smallest possible number of elements. The materials used for the envelope should provide better thermal insulation while maintaining a relatively low weight. Moreover, connections and details should enhance thermal and acoustic insulation of the structure, with limited chances of mistakes during the assembly.

#### 3.4.1. TECH 04—Design and Materials

**Figure 18:**
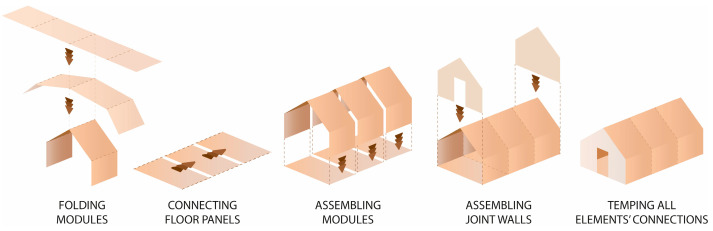
Component assembly sequence developed for the full-scale unit of TECH 04.

The design assumed a unit with an area of 10.5 m^2^ which could accommodate a family of three. The modular structure allowed the shelter to be extended by multiplication of modules with a usable area of 3.5 m^2^ (see Figure 18).

The whole structure was composed of six prefabricated elements: three envelope panels, two gable walls, and one floor panel. All the components were flat and their dimensions did not exceed 7.1 m in length and 2.5 m in width, to ensure efficient transportation. The shelter was designed for quick assembly by non-professionals, with the use of basic equipment.

**Figure 19:**
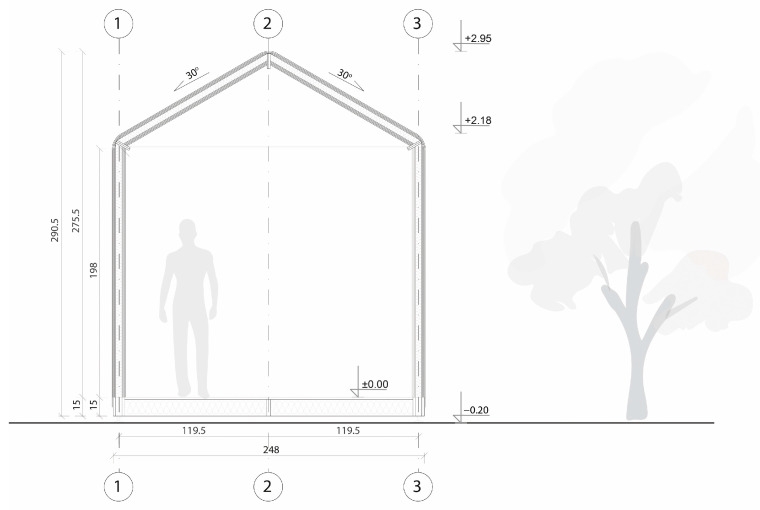
Section of TECH 04.

**Figure 20:**
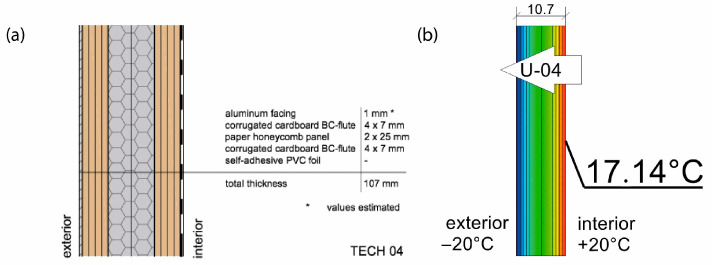
Layer composition applied in the TECH 04 external wall (**a**) and its corresponding numerical simulation of temperature distribution with indicated surface temperature (**b**).

The 11 cm thick envelope panels were composed of a double layer of 25 mm thick honeycomb panels laminated with four layers of 7 mm thick BC flute corrugated cardboard on both sides. The envelope panels were folded to form the walls and roof of the entire construction segment, as presented in Figure 19. Such a solution facilitated the assembly and ensured tightness and reduced the number of potential thermal bridges. The wooden structural elements were integrated between the layers of the envelope panels. The external surface of the panels was protected by varnish-coated aluminum roofing sheets, and polyvinyl chloride self-adhesive film was used from the inside. A PVA glue was used for paper lamination and a polyurethane-based one for aluminum lamination (see Figure 20). A back gable wall was made of the same cardboard sandwich structure with a window, while a front wall, made of translucent polycarbonate, provides daylight inside the shelter. The floor platform consisted of a timber frame structure, filled with insulative panels from laminated corrugated cardboard wrapped in polyethene foil and with OSB plates on top. Depending on the type of ground, the unit could be placed on ground screw foundations or concrete blocks.

#### 3.4.2. TECH 04—Prototyping and Assembly

**Figure 21:**
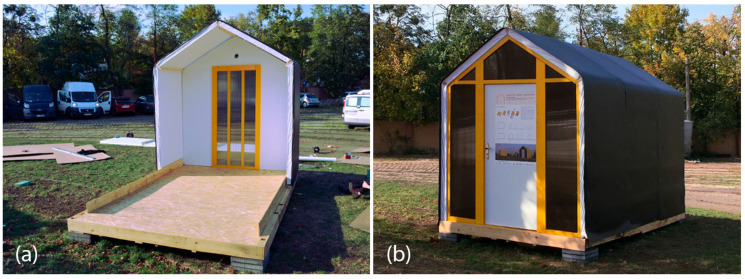
TECH 04 prototype, (**a**) assembly of subsequent segments on the timber floor, (**b**) final prototype.

The TECH 04 prototype was constructed by the researchers and a group of students in September 2018 in Wrocław, Poland. Firstly, components were prefabricated by hand in the workshop from standard paper- and wood-based materials. Envelope panels preparation was the most complex part of the work. The assembly process took one working day and the engagement of 10 people. The construction began with positioning and leveling the floor panel on the concrete blocks. Secondly, the envelope panels were folded and mounted in the slots on the floor (see Figure 21). Lastly, gable walls were attached and all the elements were screwed together. The envelope panels were joined with gasket screws through overlapping aluminum sheets. The timelapse movie of the assembly of TECH 04 can be found at: https://www.youtube.com/watch?v=gOMu-c4QLlg (accessed on 26 June 2025).

#### 3.4.3. TECH 04—Evaluation

After construction, the prototype was observed for a period of 3 years. The unit was tested in a temperate climate, in Poland. The following climatic conditions were recorded during the observation period: air temperature from −14 °C to +36 °C, relative humidity from 50% to 90%, most intense rainfall of 57 mm, and the maximum wind speed of 75 km/h.

Sufficient thermal insulation properties of the shelter were achieved thanks to a combination of corrugated cardboard and honeycomb panels. The envelope thermal conductivity was calculated as U = 0.55 W/m^2^K (for the wall). The reduction in thermal bridges improved the thermal efficiency of the whole structure—images from the thermal camera confirmed a high thermal tightness of the unit. Thanks to the specific connection between the modules, the side walls behaved as one homogenous element. The greatest heat loss occurs through the polycarbonate gable wall and at the junction of the envelope with the floor.

The metal sheet cover of the shelter significantly elongates the lifespan of the structure, ensuring excellent resistance to mechanical damage. Moreover, it increases the fire safety of the structure. Eliminating impregnation and paint coatings on cardboard also indicates the possibility of separating and recycling materials. On the other hand, the cardboard panel shrinkage during the drying process caused metal sheets deformations and a PVC foil covering the interior surface of the envelope to be prone to damage and tears easily. In the case of permanently inhabited shelter, it would be advisable to use a more durable finishing material, e.g., fiber-reinforced cladding.

The envelope modules turned out to be too heavy and too long to be easily operated. Although the structure is relatively lightweight (9.91 kg/m^2^), the whole 7.10 m × 1.20 m panel weighs approximately 85 kg. Considering its weight, length, and bendability at the grooves, handling the panels safely required the cooperation of eight people.

### 3.5. TECH 05

TECH 05 was developed as a subsequent unit intended for temporary habitation, adapted for use in a climate with distinct seasonal variation, with an expected lifespan of at least 5 years. The conceptual design was conceived in 2023 by interdisciplinary research team led by Jerzy Łątka and developed by the students of the Faculty of Architecture at Wrocław University of Science and Technology. Prefabrication of the elements took place during the international summer workshop ProtoLAB in 2023, and construction was scheduled for October 2023 in Wrocław, Poland (see Table 7).

Following the authors’ analysis and the experience from the previous four editions of TECH, a set of design criteria was established. As part of the modular assembly concept, particular attention was given to reducing the weight of individual modules while maintaining ease of assembly and design repeatability.

The design was intended for year-round use by a single family for up to five years. In this configuration, the unit is equipped with full thermal insulation, connected to basic utilities, and contains internal spatial divisions enabling it to function as a fully operational housing module.

#### 3.5.1. TECH 05—Design and Materials

The TECH 05 unit was designed as a lightweight, modular shelter intended for fast assembly and a high adaptability structure. The building consists of prefabricated panel elements, enabling transportation in a flat-pack format and simple on-site construction.

**Figure 22:**
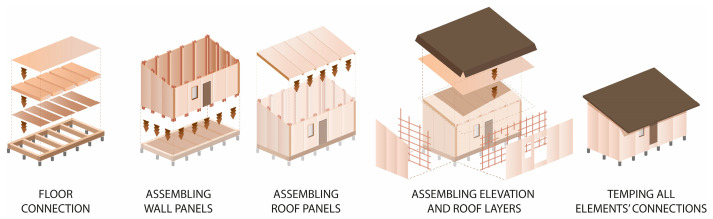
Component assembly sequence developed for the full-scale unit of TECH 05.

Each unit was designed as an independent module with a rectangular footprint and a pitched roof. The structure was composed of prefabricated wall, roof, and floor panels with integrated insulation and timber framing (see Figure 22).

**Figure 23:**
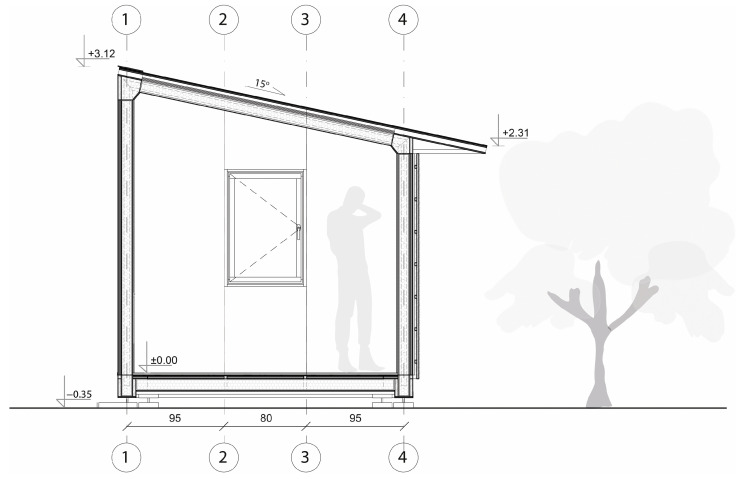
Section of TECH 05.

The roof featured a single-pitch geometry (15° slope) and was clad with a system of cardboard-based insulation and OSB boarding, topped with waterproofing layers. The modules were placed on either concrete blocks or screw foundations, depending on site conditions (see Figure 23).

Based on insights from the previous prototype, the design focused on reducing the overall weight of construction elements to enhance mobility and facilitate on-site assembly. The structure consisted of individual C-shaped wall and roof panels, made from batten framing and corrugated cardboard layers glued together. The solution was conceived to combine structural and insulative functions within the same component. The characteristic C-shape of the panels minimized thermal bridges at the connections between the walls and between roof segments.

The principal structural elements of TECH 05 consisted of wall and roof modules, designed as C-shaped components with a width of 90 cm. Each module was composed of five layers of BC-type corrugated cardboard, bonded with PVA adhesive between layers, resulting in a target thickness of 32 mm. Additionally, each panel was reinforced at critical points with softwood battens featuring a 30 mm × 30 mm cross-section. Segments of laminated corrugated cardboard were inserted between the battens to further stiffen the component, enhance its insulating performance, and maintain the module’s dimensional stability by preventing potential displacement of internal layers under load. The use of timber battens enabled the connection of individual modules using screws. This solution also allowed for the installation of a facade substructure, significantly simplifying on-site assembly and accelerating the construction process.

**Figure 24:**
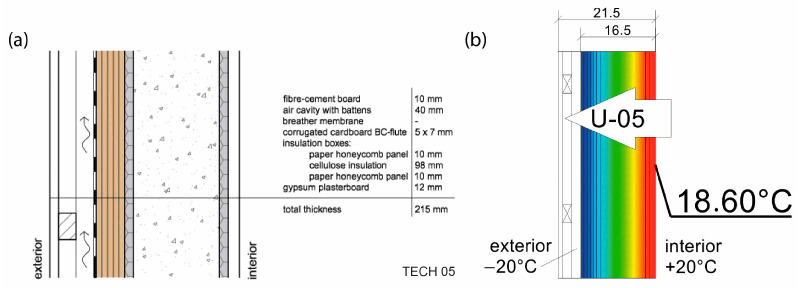
Layer composition applied in the TECH 05 external wall (**a**) and its corresponding numerical simulation of temperature distribution with indicated surface temperature (**b**).

The completed wall modules were installed on prefabricated horizontal base elements—sills made of OSBs with thicknesses of 18 mm and 12 mm. The building’s facade was designed using 10 mm thick CETRIS cement-bonded particleboard panels, mounted on a double timber subframe made of 30 mm × 40 mm battens, thus providing an external fire-resistant layer and a ventilated cavity. The roofing system consisted of a 1.5 mm thick EPDM membrane, adhered to the OSB sheathing using a dedicated polyurethane adhesive (see Figure 24).

As part of the design concept, an additional layer of thermal insulation was proposed, consisting of cardboard boxes placed within the empty cavities of the modules and filled with loose cellulose fiber. For the purposes of the constructed prototype, mineral wool insulation was applied exclusively within the floor layer.

#### 3.5.2. TECH 05—Prototyping and Assembly

The completed prototype consisted of a full-scale single unit, offering approximately 14 m^2^ of usable floor area. Prefabrication of the modules and other assembly components was carried out by students during the ProtoLAB design and build workshop under the supervision of Jerzy Łątka and Agata Jasiołek. To increase the repeatability of modules and to facilitate precise bending of cardboard along designated lines, two custom molds were developed using 18 mm MDF boards. All wall modules and majority of the assembly components were produced within two weeks from the start of the workshop, engaging approximately 13 people in the process.

Prior to transporting the structural elements to the site, preparations for the building’s foundation were initiated. The proposed foundation system consists of 12 concrete piles, each with a diameter of 20 cm and height of 80 cm, developed by using ready-mix concrete. Following a seven-day curing period, the transport of structural elements to the construction site was carried out using a medium-sized delivery van and involved a team of six people.

**Figure 25:**
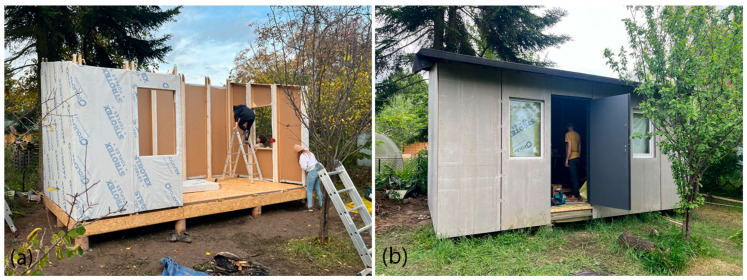
TECH 05 Prototype, (**a**) assembly of cardboard wall panels, (**b**) final prototype.

Prefabricated OSB-beams were installed on the concrete footings using chemical rods. The next stage involved fixing supporting floor beams to the sills, filling the space between them with 18 cm of mineral wool insulation, and covering the surface with OSBs that serve as the finished interior floor. Wall and roof modules followed, seated on the sills and joined with wood screws (see Figure 25a). Door and window joinery was installed directly into the wall modules using wood screws, with mounting foam used for additional support. During the foam curing period, OSBs were laid on top of the roof panels and secured with screws. Roof finishing was completed with an EPDM membrane, spray-bonded to the OSB layer using a dedicated polyurethane adhesive. The final step of the prefabrication process involved attaching a double timber substructure to the external walls, made of 30 × 40 mm battens. Cement-bonded particleboards were predrilled and mounted onto this framework using gasketed screws (see Figure 25b).

#### 3.5.3. TECH 05—Evaluation

The prototype of the TECH 05 unit was completed in November 2023 and has since been subject to ongoing observation. The prefabrication and assembly process enabled the formulation of several conclusions regarding the effectiveness of the applied structural and material solutions, as well as the identification of aspects requiring further development. The evaluation has been divided into three main categories: facade panels, the flooring system, and prefabricated structural modules.

The use of large-format 10 mm CETRIS cement-bonded particleboards, covering the entire external surface of the unit, proved problematic during both transport and installation. Due to the brittleness of the material and the large panel dimensions, handling and mounting required a team of six people, with a high risk of damage during manipulation. The process also necessitated time-consuming and precise pre-drilling of mounting holes. The construction of the complete facade, installed on a double batten substructure, demanded a high level of execution precision—both in the distribution of mounting points on the panels and in the positioning of battens within the substructure and structural wall components. Any misalignment could result in screws anchoring directly into the cardboard core rather than the timber reinforcements, significantly weakening the joints and potentially damaging the material. Achieving proper alignment between the batten spacing and the panel mounting points proved to be a highly time-consuming task, requiring close coordination. For future implementations, it is recommended to consider the use of smaller, more manageable panel formats, as well as the implementation of drilling molds, which would significantly simplify the installation process and reduce the risk of execution errors.

The use of C-shaped modules made from glued corrugated cardboard and timber battens, joined together with screws, represents a promising solution for future prototypes of similar structures. Owing to the selected materials and the geometric configuration of the elements, the resulting modules are lightweight, easy and quick to assemble, and sufficiently rigid to serve as the reliable structural system. Moreover, an additional advantage of the developed module system is the possibility of routing building services within the modular layer. This approach also prevents thermal gaps occurring at the wall–roof junctions already at the design stage and provides an additional layer of insulation, allowing for a reduction or even elimination of interior insulation boxes, depending on the intended use of the building and the required thermal performance of the envelope.

### 3.6. Design and Prototype Evaluation

The TECH project was planned as an iterative process; therefore, assessment was an essential part for each of the design and prototyping parts—those lessons learned were described in previous sections. In this section, the evaluation of the whole TECH project is presented, and versions of TECH designs are compared to each other.

#### 3.6.1. Prototyping Evaluation

First and foremost, it has been proven by prototyping process that it is possible to construct a functioning emergency shelter out of basic paper-based materials. All of the designs were prefabricated and assembled by a group of non-professionals, equipped with basic construction and carpentry tools. However, the prototyping process was more time-consuming than expected, due to manual production and the need to implement alterations to the design. Larger-scale industrial production is expected to eliminate these difficulties in real emergency scenarios. One of the most significant factors influencing the time and feasibility of the assembly process itself appeared to be the weight and size of the components. It is reasonable to design the components to the maximum size that will allow them to be comfortably lifted and carried by two people. After the construction, TECH prototypes were exposed to natural weather conditions and proved to provide sufficient resistance to water, humidity, and mechanical damage; however, they were not inhabited.

#### 3.6.2. Envelopes Evaluation

Building envelope is a crucial element of every emergency shelter. Its composition and parameters are key factors contributing to building weight, material consumption, assembly process, durability, thermal and acoustic comfort of the occupants. On the other hand, emergency scenarios often require compromises to be made between those functional properties and production cost, material availability, ease of transportation, and construction process. Therefore, a separate analysis to evaluate TECH envelopes was performed.

All of the basic envelope characteristics are summarized in Table 8. Envelopes 01–03 were lightweight sandwich panels with various configurations of honeycomb panels and protective layers of polyethylene or polyvinyl chloride foils. Those panels did not provide load-bearing properties but were fixed to internal structural frames. On the contrary, envelopes 04 and 05 are a combination of sandwich and embedded structure type, where load-bearing elements are incorporated inside the panels. In TECH 04 a finishing layer made of more durable material was introduced for the first time, providing more damage resistance and durability. Thermal properties were also improved by the addition of corrugated cardboard. In the last iteration, TECH 05, the concept was developed further, resulting in the most complex, yet more durable and insulative envelope. The ventilated air cavity was introduced for the first time to protect paper elements from moisture and extend their lifespan.

TECH envelopes represent a large variety of designs—from thin (43 mm) and exceptionally lightweight (4.93 kg/m^2^) panels of TECH 01 to more standard parameters of TECH 05 (215 mm and 41.28 kg/m^2^). All of the envelopes consist of paper-based products of more than 75% by volume, representing various levels of resistance to destructive factors, depending mostly on the share of non-paper materials in the composition.

The Performance Score achieved by envelopes is between 8 points for TECH 01 and 15 points for TECH 05 (see Table 9). This proves that successive iterations of the design have indeed improved its performance. It is also visible that higher resistance to water, fire, and mechanical damage is negatively correlated with the envelope weight, price, and material availability, as the protective layers are heavier and more expensive than paper-based products. Although TECH 05 achieved the highest thermal properties, TECH 02 is the one with the most favorable correlation between thermal properties, weight and thickness, which can largely be attributed to the lack of protective facings. The designed envelopes provide various properties that need to be evaluated considering the performance requirements of a particular area of application. The designs of TECH 01-03 may be recommended for a short-term emergency use, when low price and production time efficiency are key contributing factors. On the other hand, TECH 04 and 05 may be considered semi-permanent shelters, providing higher living standard for a longer period of time.

## 4. Conclusions

In an emergency situation, whether it is an armed conflict, political repression or natural disaster, shelter is one of the key elements of human resilience. The adequate shelter should meet the requirements of temporary housing, providing occupants with privacy, thermal comfort, and protection against weather conditions. Considering the important factor of ecological devastation caused in the local landscape, easy to recycle materials, such as paper, should be utilized for building components of relief housing units. During the development of TECH project, five types of emergency houses were designed, prototyped and evaluated. The discussed case studies present different properties and applications, varying from short-term, quick, and lightweight emergency shelters to durable semi-temporary housing.

Based on this experience, the following conclusion and guidelines for further research were formulated.

- Frame or hybrid frame-panel structural system enhance rigid and lightweight structure. Hybrid system consists of fewer components, and hence improves the assembly process, while frame system allows for replacing damaged panels during the use of the house.
- Building envelope composed of corrugated cardboard and thin honeycomb panels shows the most optimal thermal insulation and weight ratio, while maintaining structural properties of the panel.
- Thermal insulation as additive element, added to previously erected structure minimizes the weight and hence operability of the structure during transportation and assembly. This can be done as an extra outer layer or as a thermal insulation material that is inserted into the building envelope box, for example, cellulose fiber.
- Prefabrication of building components in indoor controlled conditions ensures precise manufacturing where the paper materials are not exposed to water and high humidity.
- Component sizes and weights should allow them to be comfortably handled by two people to ensure smooth assembly process.
- Use of mass-produced paper and other industries produce sub-components that are combined into building components ensures higher reproducibility and hence reduces costs.
- Water and humidity protection is crucial for paper-based structures for short lifespan foil lamination may be sufficient; however, for longer lifespans, a double-layer protection with non-paper material is recommended.
- Fire protection can be combined with the waterproof layers into a single element, made from different material.
- Building envelope ventilation—in the case of long-lasting temporary houses, the system of walls and roof ventilation is required to prevent damage caused by moisture.
- Recyclability analysis and Life Cycle Assessment, that embraces all used materials should be a part of design process, reduce environmental impact of shelters.

Since paper-based structures must meet a range of sometimes competing parameters, a systematic optimization strategy should be an essential part of the design process. Further research should focus on developing a strategy that takes into account the structural, thermal, environmental, and economic aspects of paper-based shelters.

The presented scope of further research will become aspects to be studied during creation of TECH 06, i.e., new longer lifespan version of semi-temporary housing designated for Central European climate conditions.

## Figures and Tables

**Table 1:** Parameters of the tested paper-based products.

Paper-Based Product	Description	Mechanical Parameters from Tests—Mean Values	
Paper tube 60 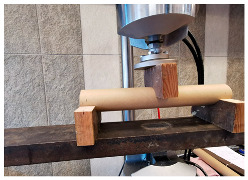	outer diameter 60 mm wall thickness 4 mm material density 650 kg/m^3^	f_c_	9.9 MPa
		f_t_	12.8 MPa
		f_m,3_	11.7 MPa
		f_m,4_	14.8 MPa
		E_t_	2.49 GPa
		E_m_	0.82 GPa
Paper tube 115 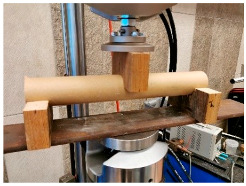	outer diameter 115 mm wall thickness 7 mm material density 650 kg/m^3^	f_c_	11.0 MPa
		f_t_	12.3 MPa
		f_m,3_	12.9 MPa
		f_m,4_	11.6 MPa
		E_t_	11.07 GPa
		E_m_	0.58 GPa
Paper tube 170 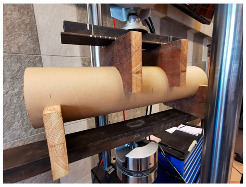	outer diameter 170 mm wall thickness 10 mm material density 650 kg/m^3^	f_c_	5.7 MPa
		f_t_	8.7 MPa
		f_m,3_	5.8 MPa
		f_m,4_	6.4 MPa
		E_t_	5.56 GPa
		E_m_	0.40 GPa
Corrugated cardboard BC flute 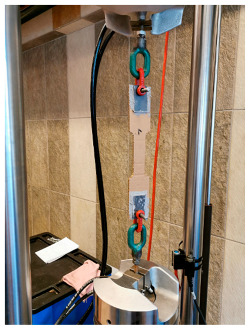	5 layers paper thickness 6.01 mm density 573 g/m^2^	f_t,par_	14.0 MPa
		f_t,per_	16.7 MPa
Honeycomb panel 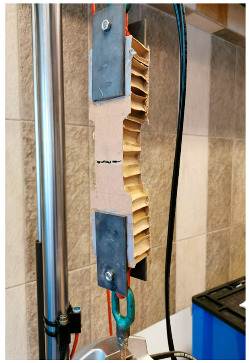	thickness 25 mm	f_t_	7.5 MPa
Honeycomb panel 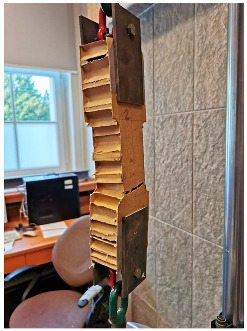	thickness 50 mm	f_t_	2.2 MPa
Rectangular paper tubes from two U-shape elements glued together 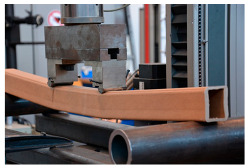	section dimensions: 58 mm × 68.5 mm wall thickness 5.5 mm element length 1080 mm	f_m,4_	6.6 MPa
		f_c_	8.3 MPa

f_c_—compressive strength, f_t_—tensile strength, f_m,3_—flexural strength (3-P bending test), f_m,4_—flexural strength (4-P bending test), E_t_—modulus of elasticity in tension, E_m_—modulus of elasticity in bending, f_t,par_—tensile strength parallel to the flute, f_t,per_—tensile strength perpendicular to the flute.

**Table 2:** Thermal conductivity of paper-based products (based on literature and own research).

Material	Type/Height/Direction	λ [W/mK]	Source
**Corrugated cardboard**	B—flute/-/Z	0.039	Own research
**Corrugated cardboard**	BC—flute/-/Z	0.044	Own research
**Corrugated cardboard**	E—flute/-/Z	0.045	Own research
**Corrugated cardboard**	EE—flute/-/Z	0.043	Own research
**Corrugated cardboard**	BC—flute/-/Y	0.090	Own research
**Corrugated cardboard**	BC—flute/-/X	0.084	Own research
**Honeycomb panel**	/10 mm/Z	0.067	Own research
**Honeycomb panel**	/25 mm/Z	0.105	Own research
**Honeycomb panel**	/50 mm/Z	0.135	Own research
**Honeycomb panel**	/25 mm/X	0.083	Own research
**Honeycomb panel**	/50 mm/X	0.074	Own research
**Cardboard**	/>1 mm/Z	0.14	[66]
**Copy paper**	/<1 mm/Z	0.25	[66]
**Corrugated cardboard**	C-flute/-/Z	0.053	[51]
**Corrugated cardboard**	E-flute/-/Z	0.058	[51]
**Corrugated cardboard**	/19 mm/Z	0.0902–0.1326	[54]
**Recycled cardboard**	/-/Z	0.09	[67]
**Corrugated cardboard**	B-flute/-/Z	0.0486	[52]
**Honeycomb panel**	/70 mm/Z	0.1167	[52]
**Honeycomb panel**	/30 mm/Z	0.0899	[52]
**Honeycomb panel**	/18 mm/Z	0.0773	[52]
**Honeycomb panel**	/13 mm/Z	0.0706	[52]
**Honeycomb panel**	/50 mm/Z	0.125	[53]
**Cardboard**	/-/Z	0.065	[56]
**Corrugated cardboard**	-/20 to 50 mm/Z	0.0477 to 0.0839	[61]
**Honeycomb panel**	/10 to 60 mm/Z	0.06 to 0.165	[68]
**Honeycomb panel**	/25 mm/Z	0.0783 to 0.1064	[69]

**Table 3:** TECH 01—prototype details and characteristics.

Prototype Details		Prototype Characteristics	
version	TECH 01	usable area	17.4 m^2^
designer	(author)	expected lifespan	18 months
year	2014	envelope U-value	1.66 W/m^2^K
place of construction	Delft, NL (only panel prototype)	structural system	hybrid rod-panel

**Table 4:** TECH 02—prototype details and characteristics.

Prototype Details		Prototype Characteristics	
version	TECH 02	usable area	13.0 m^2^
designers	Students of TU Delft	expected lifespan	3 years
year	2015	envelope U-value	0.73 W/m^2^K
place of construction	Delft, NL	structural system	rod

**Table 5:** TECH 03—prototype details and characteristics.

Prototype Details		Prototype Characteristics	
version	TECH 03	usable area	12.0; 25.0 m^2^
designers	Jerzy Łątka, Julia Schönwälder	expected lifespan	3 years
year	2016	envelope U-value	0.73 W/m^2^K
place of construction	Wrocław, PL	structural system	frame

**Table 6:** TECH 04—prototype details and characteristics.

Prototype Details		Prototype Characteristics	
version	TECH 04	usable area	13.8 m^2^
designers	Jerzy Łątka, Agata Jasiołek	expected lifespan	5 years
year	2018	envelope U-value	0.55 W/m^2^K
place of construction	Wrocław, PL	structural system	hybrid frame-panel

**Table 7:** TECH 05—prototype details and characteristics.

Prototype Details		Prototype Characteristics	
version	TECH 05	usable area	14 m^2^
designers	article authors	expected lifespan	10 years
year	2024	envelope U-value	0.27 W/m^2^K
place of construction	Wrocław, PL	structural system	hybrid frame-panel

**Table 8:** Characteristics and comparison of TECH envelopes.

		TECH 01	TECH 02	TECH 03	TECH 04	TECH 05
envelope structure type		sandwich	sandwich	sandwich	sandwich/embedded	sandwich/embedded
expected lifespan		18 months	3 years	3 years	5 years	10 years
load-bearing		no	no	no	yes	yes
thickness (d) [cm]		4.3	8.8	15.0	10.7	21.5
ventilation		no	no	no	no	yes
weight (m) [kg/m^2^]		4.93	6.09	5.83	9.91	41.28
heat of combustion [MJ/m^2^]		104.95	126.85	122.95	172.35	144.65
thermal transmittance (U) [W/m^2^K]		1.66	0.73	0.73	0.55	0.27
thermal resistance (R) [m^2^K/W]		0.60	1.37	1.37	1.82	3.70
R:d ratio		0.14	0.16	0.09	0.17	0.17
R:m ratio		0.12	0.22	0.23	0.18	0.09
paper share, by weight		91%	90%	93%	75%	20%
paper share, by volume		99%	98%	99%	98%	76%
insulative core material		HP	CC, HP	HP	CC	CC, CF
internal surface	protection technique	FL	-	2× FL	FL	AM
	fire resistance	low	low	low	low	high
	water resistance	medium	low	medium	medium	medium
	mechanical resistance	low	low	low	low	high
external surface	protection technique	FL	FL	2× FL	AM	FL, AM
	fire resistance	low	low	low	medium	high
	water resistance	medium	medium	medium	high	high
	mechanical resistance	medium	low	low	high	high
price and material availability		good	good	good	medium	poor

HP—honeycomb panel, CC—corrugated cardboard, CF—cellulose fiber, FL—foil lamination, AM—additional material.

**Table 9:** Performance score of TECH envelopes.

	TECH 01	TECH 02	TECH 03	TECH 04	TECH 05
U [W/m^2^K]	0	1	1	1	2
R:d ratio	1	2	0	2	2
R:m ratio	0	2	2	1	0
fire resistance	0	0	0	1	4
water resistance to water	2	1	2	3	3
mechanical resistance	1	0	0	2	4
price and material availability	4	4	4	2	0
**Performance Score**	**8**	**10**	**9**	**12**	**15**

## Data Availability

The original contributions presented in this study are included in the article. Further inquiries can be directed to the corresponding author.
